# A prospective phase I study of hypo-fractionated neoadjuvant radiotherapy for locally advanced gastric cancer

**DOI:** 10.1186/s12885-018-4707-9

**Published:** 2018-08-08

**Authors:** Ning Li, Xin Wang, Yuan Tang, Dongbing Zhao, Yihebali Chi, Lin Yang, Liming Jiang, Jun Jiang, Wenyang Liu, Yu Tang, Hui Fang, Yueping Liu, Yongwen Song, Shulian Wang, Jing Jin, Yexiong Li

**Affiliations:** 1 Department of Radiation Oncology, Chinese National Cancer Center/Cancer Hospital, Chinese Academy of Medical Sciences, 17 Panjiayuannanli, Chaoyang District, Beijing, 100021 China; 2 Department of Abdominal Surgical Oncology, Chinese National Cancer Center/Cancer Hospital, Chinese Academy of Medical Sciences, Beijing, China; 3 Department of Medical Oncology, Chinese National Cancer Center/Cancer Hospital, Chinese Academy of Medical Sciences, Beijing, China; 4 Department of Radiology, Chinese National Cancer Center/Cancer Hospital, Chinese Academy of Medical Sciences, Beijing, China

**Keywords:** Gastric cancer, Chemoradiotherapy, Phase I study, Hypo-fractionated radiotherapy

## Abstract

**Background:**

Previous studies have reported that neoadjuvant chemoradiotherapy can downstage the advanced gastric cancer. However, no studies are available on the application of hypo-radiotherapy to neoadjuvant radiotherapy. This study sought to assess the maximum tolerated dose (MTD) and dose-limited toxicity (DLT) of hypo-fractionated chemoradiotherapy for local advanced gastric cancer.

**Method:**

Patients with cT3–4 and/or lymph node-positive locally advanced gastric cancer or Siewert II/III esophagogastric junction adenocarcinoma were enrolled. Preoperative chemoradiation was followed by 3 cycles of oxaliplatin + S-1 neoadjuvant chemotherapy with an interval duration of 3–4 weeks. D2 resection was performed 2–4 weeks after neoadjuvant therapy. Three cycles of adjuvant chemotherapy were planned after surgery. Intensity-modulated radiotherapy (IMRT) was used. The radiotherapy dose level was defined using three levels, namely, 40.0 Gy/2.5 Gy, 41.6 Gy/2.6 Gy, 43.2 Gy/2.7 Gy delivered concurrently with S-1 at 80 mg/m^2^.

**Results:**

From May 2016 to Dec 2016, nine patients with a median age of 63 years were enrolled in this study. The most common grade I-III adverse events were leukopenia (88.9%), nausea (88.9%), vomiting (77.8%) and weight loss (66.7%). Grade III adverse events consisted of vomiting and weight loss.

**Conclusion:**

The MTD of hypo-fractionated radiotherapy for locally advanced gastric cancer was 40.0 Gy/2.5 Gy, and the DLTs were vomiting and weight loss.

**Trial registration:**

Clinicaltrials.gov ID: NCT03427684 (Retrospectively registered on February 9, 2018).

## Background

Gastric cancer is one of the most common malignant tumors in China, and the incidence and mortality rates for this disease rank second among all malignant tumors [1]. Gastric cancer requires integrated multidisciplinary treatment, and surgery is currently the only possible curative method. Previous studies have reported that neoadjuvant chemoradiotherapy can downstage the primary tumor to increase the radical resection rate for improved long-term prognosis of advanced gastric cancer [2–5]. However, no studies are available on the application of hypo-radiotherapy to neoadjuvant radiotherapy in gastric cancer. The aim of this study was to observe the maximum tolerated dose (MTD) and dose-limited toxicity (DLT) of hypo-fractionated radiotherapy for locally advanced gastric cancer.

## Methods

### Inclusion criteria

Clinical stage T3–4 N + M0 gastric cancer or Siewert II/III esophagogastric junction carcinoma; pathologically confirmed adenocarcinoma; 18–75 years old, male or female; Karnofsky score ≥ 70; white blood cell count ≥4 × 10^9^/L; platelet count ≥100 × 10^9^/L; serum creatinine ≤1× upper limit of normal, total bilirubin ≤1× upper limit of normal, alanine aminotransferase and aspartate aminotransferase ≤2.5× upper limit of normal, and alkaline phosphatase ≤5× upper limit of normal.

### Treatment procedure

**Fig. 1:**
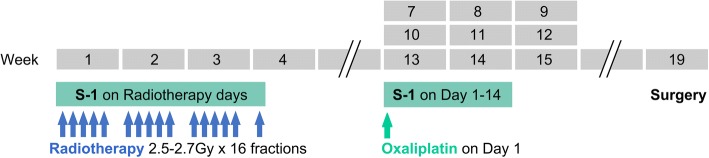
Treatment schedule

After enrollment in the study, patients were first treated with radiotherapy concurrent with oral S-1 at 80 mg/m^2^/day on radiotherapy days. Three weeks after the end of radiotherapy, patients were treated with neoadjuvant chemotherapy with oxaliplatin and S-1 (SOX) for 3 cycles. Oxaliplatin was given at a dose of 130 mg/m^2^ intravenous (iv) on day 1, and S-1 at 40–60 mg orally BID was given on days 1–14. Imaging evaluation was performed 3 weeks after neoadjuvant treatment (Fig. 1). The radical operation and surgical procedures were determined based on multidisciplinary team (MDT) discussion. Non-operable patients continued with 3 cycles of chemotherapy, and the chemotherapy regimen could be changed. Three cycles of SOX adjuvant chemotherapy were performed after surgery.

### Radiotherapy and dose escalation method

**Fig. 2:**
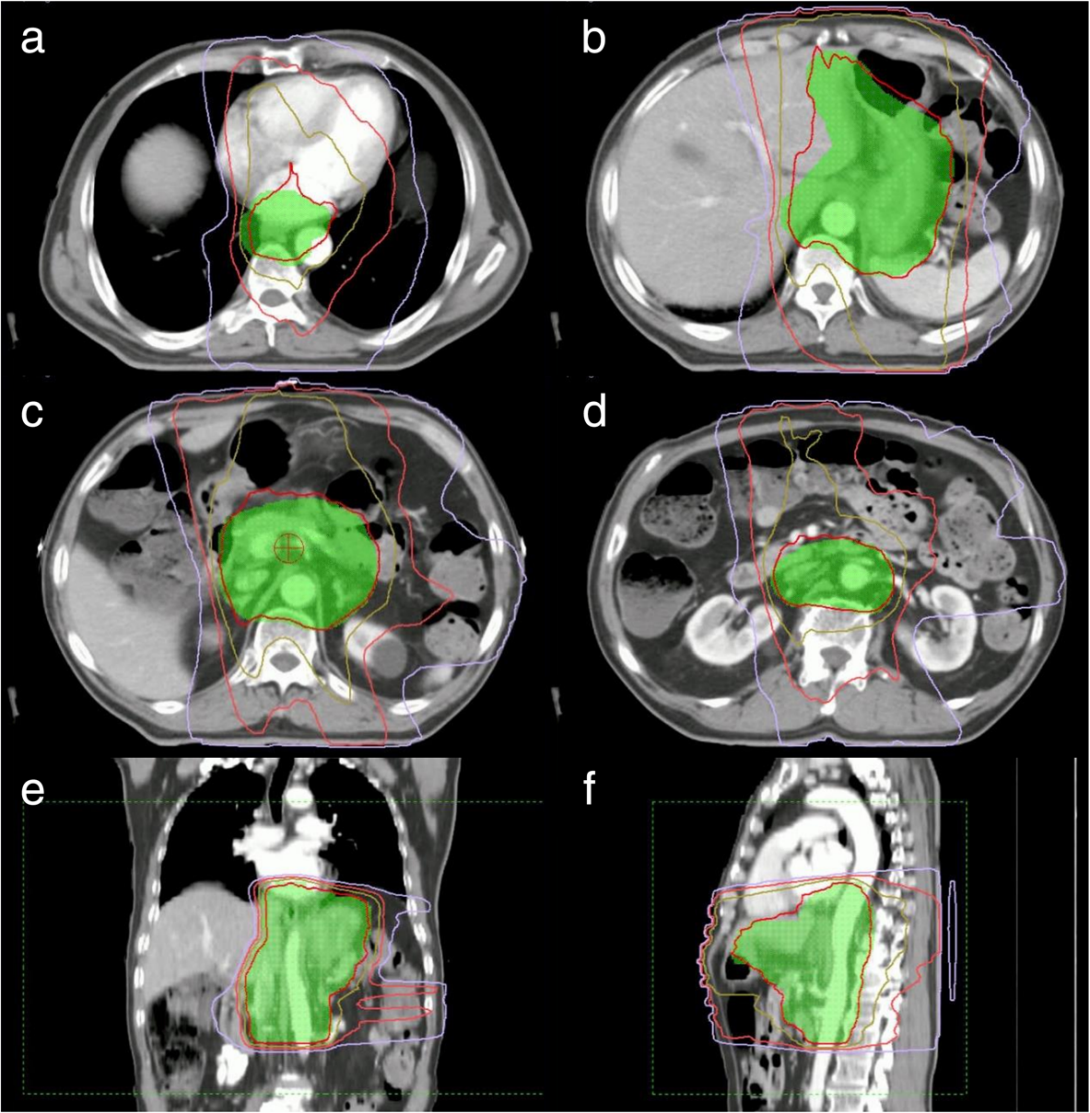
Dose distributions of 250 cGy × 16 fractions radiotherapy plan with 6MV-X based volumetric-modulated arc radiotherapy (VMAT) on **a** top level, **b** GTV level, **c** center level, **d** bottom level, **e** sagittal view and **f** coronal view. Isodose line of 1000 cGy, 2000 cGy, 3000 cGy, 4000 cGy and 4400 cGy were colored in purple, rose red, gold, red and yellow, respectively

The patients were fasting for more than 4 h before positioning, and a CT scan was performed after body film fixation. According to gastroscopy, MRI and CT, we determined the GTV range of primary tumors and lymph nodes. CTV includes GTV with 2.5 cm expanded in the mucosal direction and GTVnd. According to the location of the primary tumor, CTV included elective lymph nodes (LNs) regions (Table 1). Peri-gastric lymph node regions without GTVnd were excluded from the CTV. PTV was based on 7 mm radial and 10 mm proximal and distal expansions from CTV. The whole lung, liver, kidney, heart and spinal cord were contoured as normal tissue. The plan met the dose constraints to critical organs. The maximum dose of spinal cord could not exceed 45 Gy; lung V20 < 20%, V5 of the entire lung should have been less than 2300 CC; kidney V20 < 30%; cardiac V40 < 30%, V25 < 50%; liver V30 < 30%; small intestine V15 < 275 CC, V40 < 150 CC. Intensity-modulated radiotherapy (IMRT), or volumetric-modulated arc radiotherapy (VMAT) technique was used (Fig. 2). We used the image-guided radiotherapy at the same time to ensure the accuracy of the position.

**Table 1:** Post-operative radiation elective lymph node regions

Primary tumor segment	Elective lymph nodes regions
Siewert II/III or proximal gastric cancer	7,8,9,11p,16a2,16b1^b^
Middle or distal gastric cancer	7,8,9,11p,12a,13,14^a^,16a2,16b1^b^

^a^If the lymph node metastases in LN station No.6, CTV should encompass LN station No.14

^b^If the lymph node metastases in LN station No.7–12, or stage N2/3, the lower border of CTV should encompass LN station No.16b1

DLT was defined as the presence of the following adverse events during radiotherapy and within 1 month after the end of radiotherapy: ≥ grade 4 myelosuppression (leukopenia or thrombocytopenia), ≥ grade 3 non-blood adverse events (such as nausea, vomiting, fatigue, fever, pain, weight loss and other symptoms associated with treatment), and ≥ grade 2 abnormal renal and liver function. The dose of radiotherapy was increased progressively as follows: reference dose 40 Gy/2.5 Gy/16 fractions, 95% isodose line covering the planning target volume (PTV); 95% PTV 41.6 Gy/2.6 Gy/16 fractions; and 95% PTV 43.2 Gy/2.7 Gy/16 fractions. At least 3 patients were treated in each dose group. If DLT was not found in 3 patients, then the step to the next dose level was applied until the DLT occurred. If more than 2 DLTs occurred, the dose was the DLT dose level. If a dose group showed 1 case of DLT in the first 3 cases, the other 3 cases were added to the dose level. If the additional 3 cases did not incur DLT, the dose level was escalated to a higher level. If one or more DLTs appeared in the additional 3 cases, the dose level was the MTD level. If the above two circumstances appeared, the dose escalation was terminated, and the previous dose level was chosen as the MTD, which is also the recommended dose for a phase II clinical study.

### Preoperative assessment and postoperative pathological evaluation

Preoperative TNM staging was evaluated via thoracic, abdominal and pelvic CT, gastroscopy, endoscopic ultrasonography and gastric MRI. PET scan and diagnostic laparoscopy were not mandatory. Surgical resection specimens were subjected to the Mandard tumor regression grade (TRG) classification standard to conduct an overall evaluation of primary lesions and lymph nodes. Grade 1 indicated complete regression; grade 2 showed fibrotic tissue with a small amount of residual cancer cells; grade 3 was assigned when cancer cells were present, but the residual fibrosis was dominant; grade 4 showed a greater proportion of residual cancer cells over fiber degeneration; and grade 5 indicated no signs of regression [6].

### Patient follow-up

Follow-up occurred at 3-month intervals for 2 years, then at 6-month intervals until 5 years. Diagnostic evaluations were performed using CT of the chest and abdomen, and MRI or gastroscopy only if necessary.

### Statistical method

The Kaplan-Meier method was used to calculate survival rate using SPSS 22.0 software (IBM, New Orchard Road Armonk, New York 10,504). The survival calculation was determined from the date of enrollment to death or to the last follow-up visit.

## Results

### Patient characteristics

From May 2016 to Dec 2016, 9 patients provided written consent. All the enrolled patients were male, and the median age was 63 years old (53–67). The Karnofsky scores of 8 cases were 90, and 1 case had a score of 80. There were 4 cases of Siewert II/III esophagogastric junction carcinoma and 5 cases of gastric cancer. There were 4 cases in stage IIIA, 2 in stage IIIB, 1 in stage IIIC and 2 in stage IIB (Table 2).

**Table 2:** Patient characteristics

	Number (Percent)
Median age (Range)	63(53–67)
Gender	
Male	9(100.0)
KPS score	
90	8(88.9)
80	1(11.1)
Segment	
Siewert II/III	4(44.4)
Gastric	5(55.6)
T stage	
T3	1(11.1)
T4a	6(66.7)
T4b	2(22.2)
N stage	
N0	1(11.1)
N1	6(66.7)
N2	2(22.2)
Clinical stage	
IIB	2(22.2)
IIIA	4(44.4)
IIIB	2(22.2)
IIIC	1(11.1)

### Radiotherapy dose escalation results

Of the 9 patients who completed the 2-level dose escalation trial, the 40 Gy/2.5 Gy group did not exhibit any DLTs. In the first 3 cases at the 41.6 Gy/2.6 Gy dose level, DLT occurred in 1 patient (grade 3 gastrointestinal toxicity), and 1 DLT occurred in an additional 3 cases (grade 3 weight loss) (Table 3). Therefore, 41.6 Gy/2.6 Gy was the DLT dose, and 40 Gy/2.5 Gy was the MTD dose.

**Table 3:** Dose-limited toxicity

Dose level	Dose	n	Number of DLT	DLT	Radiotherapy dose when DLT
1	40 Gy/2.5 Gy	3	0	–	–
2	41.6 Gy/2.6 Gy	6	2	Vomiting	31.2 Gy
				Weight loss	41.6 Gy

### Side effects

The most common grade 1–3 adverse events were leukopenia (88.9%), nausea (88.9%), vomiting (77.8%) and weight loss (66.7%). The toxicity at grade 3 consisted of vomiting and weight loss (Table 4). Of the 9 cases, one patient’s concurrent chemoradiotherapy was interrupted for 7 days due to grade 3 vomiting.

**Table 4:** Toxicity incidence of hypo-fractionation radiotherapy for gastric cancer [n (%)]

	Grade 0	Grade 1	Grade 2	Grade 3	Grade 4
Leukopenia	1(11.1)	7(77.8)	1(11.1)	0(0)	0(0)
Anemia	8(88.9)	1(11.1)	2(22.2)	0(0)	0(0)
Thrombocytopenia	1(11.1)	1(11.1)	2(22.2)	0(0)	0(0)
ALT	9(100)	0(0)	0(0)	0(0)	0(0)
AST	9(100)	0(0)	0(0)	0(0)	0(0)
TBIL	9(100)	0(0)	0(0)	0(0)	0(0)
Nausea	1(11.1)	4(44.4)	4(44.4)	0(0)	0(0)
Vomiting	2(22.2)	3(33.3)	3(33.3)	1(11.1)	0(0)
Diarrhea	7(77.8)	2(22.2)	0(0)	0(0)	0(0)
Fatigue	8(88.9)	0(0)	1(11.1)	0(0)	0(0)
Weight loss	3(33.3)	3(33.3)	2(22.2)	1(11.1)	0(0)

### Completion of treatment

All the patients completed concurrent chemoradiotherapy, and one patient refused any other treatments after concurrent chemoradiotherapy. The other 8 patients received 3 cycles of neoadjuvant chemotherapy. The locoregional clinical response rate for the neo-adjuvant treatment was 100% (complete response rate = 22.2%, partial response rate = 77.8%). Four patients underwent radical surgery. Of the 5 patients who did not receive surgery, 1 patient refused the operation. The other 4 patients were unable to undergo the operation because of distant metastasis, and the alternative chemotherapy regimen was given for systemic therapy.

### Short-term efficacy and survival

The postoperative pathological complete response (pCR) rate in the surgery group was 50%, with pathological stage 0 in 2 cases, stage IIa in 1 case, and stage IIIa in 1 case. TRG grades 1, 2 and 3 consisted of 2 cases, 1 case and 1 case, respectively. The median follow-up time was 17 months. The median time of disease-free survival was 16.6 months. Four patients who received surgery and 1 patient who refused surgery showed no recurrence or metastasis at the end of the follow-up period. Of the 4 patients with distant metastasis, 2 had liver metastases, and 2 had peritoneum metastases.

## Discussion

The main purpose of this study was to observe the MTD and DLT of hypo-fractionated radiotherapy for local advanced gastric cancer. The results showed that DLTs occurred at the 41.6 Gy/2.6 Gy dose level; thus, we identified 40 Gy/2.5 Gy as the MTD dose. The most common side effects of grade 1–3 were leucopenia, nausea, vomiting and weight loss. DLTs consisted of grade 3 vomiting and weight loss.

The MAGIC and FNCLCC/FFCD studies identified the therapeutic modalities of preoperative chemotherapy for gastric cancer [7, 8]. The short-term response rate of radiotherapy and local regional control present obvious advantages. The phase 3 randomized controlled study from our center compared the prognosis of preoperative radiotherapy with that of surgery alone. The preoperative radiotherapy group received a 40 Gy dose of radiotherapy prior to surgery. The results showed that the 5-year and 10-year overall survival rates in the preoperative radiotherapy group were 30.1 and 19.75%, respectively, which were significantly better than those in the surgery alone group (20.3 and 13.3%, respectively; *p* = 0.009) [9]. In the CROSS study, a similar conclusion was obtained [5]. In recent years, total neo-adjuvant treatment has become a topic of high interest in the treatment of gastric cancer. In Stahl’s study, chemotherapy and radiotherapy were performed as neoadjuvant therapy, and the long-term prognosis was discussed. Three hundred and fifty-four clinical stage T3–4NanyM0 esophageal and gastric adenocarcinoma patients were enrolled. The control group with cisplatin plus 5-fluorouracil and leucovorin (PLF) underwent surgery, and the experimental group received radiotherapy over 3 weeks concurrent with the same chemotherapy regimen followed by surgery. No postoperative adjuvant therapy was given. Although the toxicity of the preoperative chemoradiotherapy group increased (grade 3 to 4 toxicity: 12% vs. 5%), chemoradiotherapy significantly improved the pCR rate (15.6% vs. 2%) and the pathologic N0 rate (64.4% vs. 37.7%). The five-year overall survival rate was higher in the chemoradiotherapy group (39.5% vs. 24.4%, *p* = 0.055) [3]. Although the study only recruited 126 patients due to slow recruiting speed, and the research results revealed no significant differences in overall survival, the results showed that preoperative chemoradiotherapy could improve the patient survival trend for locally advanced adenocarcinoma of the esophagogastric junction. The RTOG9904 study reported by Maurel was a single-arm phase 2 study. All the recruited patients received radiotherapy concurrent with chemotherapy (5-fluorouracil and paclitaxel) after 2 cycles of PLF chemotherapy. Radical surgery was performed 5 to 6 weeks after the neoadjuvant treatment, and no adjuvant chemotherapy was given. The study included 49 patients, and the pCR rate and R0 resection rate were 26 and 77%, respectively. The grade 4 toxicity rate was 21% [10]. These previous clinical studies of neo-adjuvant chemoradiotherapy showed that this treatment modality could improve the pCR rate and improve the long-term outcome. Our study examined the therapeutic modalities of concurrent chemoradiotherapy and perioperative chemotherapy plus radical surgery. The pCR rate was 50% in the patients who underwent surgery. One patient received no further treatment after concurrent chemoradiotherapy, and this patient is currently in a state of disease-free survival. Therefore, the effectiveness of the total neo-adjuvant treatment modality warrants further study.

The conventional radiotherapy fraction mode is routinely used in gastric cancer. Selected studies have attempted hypo-fractionated treatment for palliative radiotherapy [11–15]. A total of 107 patients were enrolled in the study by Tey et al., in which the radiotherapy dose ranged from single 8 Gy to 40 Gy/16 f [15]. The entire stomach was irradiated using the AP-PA radiation technique, and the results showed that the incidence of grade 3 toxicity in the entire group was 3.8%, consisting of gastrointestinal toxicities. No grade 4 or 5 toxicity occurred; therefore, the technique was considered tolerable for hypo-fractionated radiotherapy. Compared with the above studies, we used 2.5–2.7 Gy as a fraction to escalate the dose. Our target volume did not include the entire stomach, and the radiotherapy plan was performed using IMRT technology. Therefore, we believe that the fraction mode is safe and tolerable. Additionally, hypo-fractionated radiotherapy has the advantage of shortening the total neoadjuvant treatment duration and saving medical resources. Our results showed that 41.6 Gy/2.6 Gy was the DLT dose level, and the major toxicities included gastrointestinal adverse events and weight loss. No serious complications occurred during or after surgery.

In the perioperative treatment of gastric cancer, S-1 has been widely used in Asia [16, 17]. The previous phase I study from our center assessed the MTD of S-1 in postoperative chemoradiotherapy for gastric cancer [18]. The conventional fraction radiotherapy dose of 45 Gy/1.8 Gy was delivered, and the MTD was 80 mg/m^2^. The same concurrent chemoradiotherapy regimen was also used in the phase II study of preoperative gastric cancer with concurrent chemoradiotherapy. Preliminary results showed that the tolerances of radiotherapy and concurrent chemotherapy reached 96.7 and 93.3%, respectively [19]. However, this scheme has not been widely used in Western white populations. Several studies have reported that the level of the CYP2A6 enzyme is higher in the white population than in the Asian population, such that the maximum tolerance dose is lower [20, 21]. The current study fixed the S-1 dose to 80 mg/m^2^, as recommended by the previous study, and the design was planned to escalate the hypo-fractionated radiotherapy dose.

In this study, a new modality of radiotherapy and chemotherapy for local advanced gastric cancer was discussed, thus offering a foundation for further research. However, this study observed worse prognoses than those reported in the literature, and we attempted to analyze the underlying reasons. First, the overall clinical staging of the enrolled patients was late. Seven cases were graded as stage III, and the prognosis for these patients was poor. Second, 2 patients had peritoneal metastasis shortly after chemoradiotherapy. According to past reports, the incidence of peritoneal metastasis in gastric cancer is as high as 9.8–19.3% [22, 23], and peritoneal metastasis is difficult to diagnose prior to surgery. Therefore, peritoneal metastasis is often a factor that influences staging bias and leads to poor prognosis. In addition, this study was designed for phase I clinical studies, and the sample size might have interfered with the outcome of the final prognosis. Therefore, in further large-sample studies, we intend to design additional clinical means for more accurate staging and reduced data bias.

## Conclusion

In summary, hypo-fractionated radiotherapy for local advanced gastric cancer is safe and feasible and is well tolerated. The recommended dose of radiotherapy is 40 Gy/2.5 Gy. The DLTs included gastrointestinal toxicity and weight loss. Further evaluation of the response rate and long-term outcome remains to be observed in large-sample studies.

## Abbreviations

DLT: Dose-limited toxicity
MDT: Multidisciplinary team
MTD: Maximum tolerated dose
pCR: Pathological complete response
PTV: Planning target volume
TRG: Tumor regression grade
